# Germline reflex *BRCA1/2* testing following tumor-only comprehensive genomic profiling: why, when, and how

**DOI:** 10.1093/jncics/pkae096

**Published:** 2024-10-03

**Authors:** Giulia Maneri, Camilla Nero, Luciano Giacò, Giovanni Scambia, Angelo Minucci

**Affiliations:** Departmental Unit of Molecular and Genomic Diagnostics, Fondazione Policlinico Universitario A. Gemelli IRCCS, Rome, Italy; Genomics Research Core Facility, Gemelli Science and Technology Park (GSTeP), Scientific Directorate, IRCCS Fondazione Policlinico Universitario Agostino Gemelli, Rome, Italy; Department of Woman, Child and Public Health, Fondazione Policlinico Universitario A. Gemelli IRCCS, Roma, Italy; Bioinformatics Research Core Facility, Gemelli Science and Technology Park (GSTeP), Scientific Directorate, Policlinico Gemelli IRCCS Foundation, Rome, Italy; Department of Woman, Child and Public Health, Fondazione Policlinico Universitario A. Gemelli IRCCS, Roma, Italy; Institute of Obstetrics and Gynecology, Catholic University of the Sacred Heart, Rome, Italy; Departmental Unit of Molecular and Genomic Diagnostics, Fondazione Policlinico Universitario A. Gemelli IRCCS, Rome, Italy; Genomics Research Core Facility, Gemelli Science and Technology Park (GSTeP), Scientific Directorate, IRCCS Fondazione Policlinico Universitario Agostino Gemelli, Rome, Italy

## Abstract

The majority of tumor comprehensive genomic profiling (CGP) currently does not include a matched normal control. The use of a tumor-only CGP approach needs the development of a strategy to refine germline pathogenic/likely pathogenic variants (gP/LPVs) calls, so as to limit the performance of unnecessary germline reflex tests and instead successfully identify patients who are carriers of likely gP/LPVs. Guidelines have been developed for the identification of gP/LPVs in BRCA1/2 genes on the basis of tumor-only CGP results and for the evaluation of the appropriateness of performing germline reflex BRCA1/2 testing. In this study, an algorithm to assist decision-making for germline reflex testing of BRCA1/2 variants following tumor-only CGP is proposed.

The clinical care of individuals with cancer can include tumor sequencing by comprehensive genomic profiling (1). Although the main goal of comprehensive genomic profiling is to detect actionable alterations, it can also identify germline pathogenic or likely pathogenic variants within the so-called “cancer susceptibility genes,” such as *BRCA1/2* (*BRCA*) genes (2). Somatic and germline *BRCA* alterations play a role in guiding therapeutic options (3). It is critical, however, to report, then further investigate potentially germline pathogenic or likely pathogenic variants identified in tumor-only sequencing, given the additional and substantial clinical implications for patients and their families. Comprehensive genomic profiling is becoming increasingly widespread across many referral oncology centers, but managing incidental germline findings remains challenging and is not always clearly defined. In this regard, current guidelines report slightly different and complementary recommendations.

The National Comprehensive Cancer Network recommends confirmatory testing for *BRCA* pathogenic or likely pathogenic variants, even though these variants can often be inferred with a high degree of confidence through tissue profiling. The decision to pursue confirmatory testing should be guided by the patient’s medical history, clinical characteristics, and (in some cases) the frequency of the pathogenic or likely pathogenic variants (4).

In 2019, the European Society for Medical Oncology Precision Medicine Working Group developed recommendations to optimize the detection of true potentially germline pathogenic or likely pathogenic variants in comprehensive genomic profiling. The Precision Medicine Working Group found that crude “pan-tumor” variant allele frequency thresholds (20% for small insertions and deletions, 30% for single-nucleotide variants [formerly single-nucleotide polymorphisms]) resulted in a substantial reduction of almost half the number of tumor-detected variants requiring follow-up, with a minimum loss of true germline variants (3.5%) and even smaller proportion of potentially germline pathogenic variants in comprehensive genomic profiling (1.7%) (2).

Finally, a multidisciplinary American Society of Clinical Oncology guideline staff panel developed a guideline based on a systematic review and reached a consensus on a hybrid version of the European Society for Medical Oncology Precision Medicine Working Group’s approach. The expert panel pointed out that variant allele frequency is not on its own sufficient to confirm or exclude a germline origin and that other factors (eg, tumor purity) must be considered. Furthermore, germline confirmatory tests for founder variations should be performed regardless of the patient’s ancestry or tumor type (5). Recently, Klek et al. (6) demonstrated that involvement of clinical geneticists in a tumor-only sequencing data review process can improve the identification of patients and families with pathogenic or likely pathogenic variants in comprehensive genomic profiling.

In general, variant allele frequencies close to 50% and 100% are assumed to represent heterozygous or homozygous germline pathogenic variants, respectively, and lower variant allele frequencies are predicted to be associated with somatic mutations. It is important to note that compared with hybrid capture–based next-generation sequencing strategies, polymerase chain reaction (PCR)–based next-generation sequencing strategies may not yield as consistent a variant allele frequency, especially in formalin-fixed, paraffin-embedded samples with poor DNA quality and quantity (2).

In addition, the absolute variant allele frequency of alterations from next-generation sequencing may be also affected by their genomic position or their size, such that those variants located in regions of high homology or variants comprising large insertions and deletions may have lower-than-expected coverage in the total mapped reads. This method is challenged by variable tumor heterogeneity; normal tissue contamination; and, most notably, variant allele frequency fluctuations due to copy number alteration events (7).

In support of the latter evidence, Hayashi et al. (8) reported a peculiar case of uterine sarcoma with the pathogenic *BRCA2* p.(Ile2675Val) variant (present in the ClinVar database with 17 entries) with low variant allele frequency (5%) identified in tumor-only comprehensive genomic profiling that was then confirmed to be germline in matched tumor-normal comprehensive genomic profiling. The tumor cell content in the sample was 95%. The authors explained this evidence by reporting that when genomic loss occurred at the ipsilateral allele, which carries the germline variant in tumor cells, the variant allele frequency of the germline variant was 5% (calculated as 100% normal tissue minus 95% tumor content), indicating that the sequencing predominantly captured normal tissue.

Genetic susceptibility of *BRCA* germline pathogenic variants is well established for breast, ovarian, prostate, and pancreatic cancers, while it remains unclear for uterine sarcoma. This last sentence could explain the behavior of the *BRCA2* gene, missing the mutated allele as a “somatic second hit” in the Hayashi case.

In this context, we reported the case of a 57-year-old woman diagnosed with ovarian cancer and profiled within a comprehensive genomic profiling institutional program (ClinicalTrials.gov identifier NCT06020625) (1). A *BRCA2* c.3860del, p.(Asn1287IlefsTer6) variant (known to be a germline variant and present in ClinVar with 24 entries) was identified by tumor-only comprehensive genomic profiling, with a variant allele frequency of 20.7%. Again, data analysis showed a *BRCA2* loss, and the tumor cell content was 85%. Blood testing confirmed the germline origin. In this case, it is interesting to note the behavior of the *BRCA2* gene in an oncogene-addicted tumor. Consistently, assessment of homologous recombination deficiency identified homologous recombination deficiency positivity.

Reflex *BRCA* testing can be performed by targeted Sanger sequencing or by whole *BRCA* next-generation sequencing, given the reduced costs of next-generation sequencing, which are now almost comparable to those of Sanger, with the opportunity to overcome the Sanger method’s pitfalls (11). Matched tumor-normal comprehensive genomic profiling is emerging as a strategy to provide an integrated approach for somatic and germline analyses.

In conclusion, when *BRCA* pathogenic variants are identified in tumor-only comprehensive genomic profiling, we must consider the possibility that the detected variants could have germline origins, even if their variant allele frequency is low—either near or below the established cutoff—regardless of the tumor analyzed, especially if the identified variant is known to be germline (present in the germline database [eg, ClinVar]) and genomic loss and high tumor cell content are also observed.

An algorithm based on the current guidelines and evidence reported in this article to assist decision making for germline reflex testing of *BRCA* variants detected by tumor-only comprehensive genomic profiling is proposed (Figure 1).

**Figure 1.**
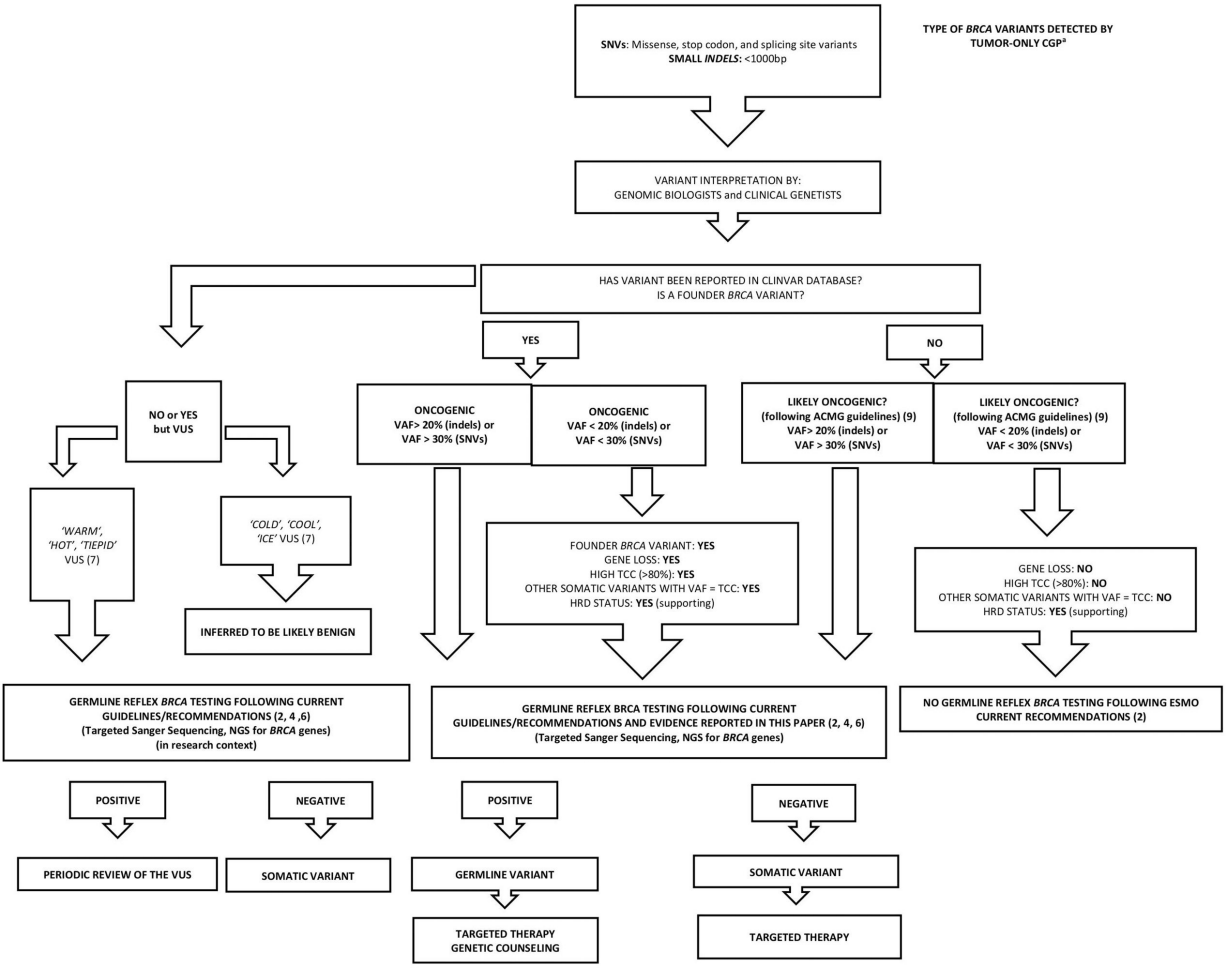
Algorithm to assist decision making for germline reflex testing of *BRCA* variants detected by tumor-only comprehensive genomic profiling. This algorithm takes into account current guidelines and recommendations (National Comprehensive Cancer Network, European Society for Medical Oncology, American Society of Clinical Oncology) and the evidence reported in this article that could be useful in routine clinical practice for oncologists and other physicians to appropriately and decisively refer to germinal reflex testing only patients with *BRCA* pathogenic or likely pathogenic variants or variants of uncertain significance, detected by tumor-only comprehensive genomic profiling, who meet specific characteristics. ^a^ Intragenic *BRCA* copy number alterations are excluded because of the need for a dedicated decision-making algorithm. VUS = Variant of Uncertain Significance; ACMG = American College of Medical Genetics and Genomics; SNVs = Single Nucleotide Variants; VAF = Variant Allele Frequency; BRCA = BRCA1/2; NGS = Next Generation Sequencing; TCC = Tumor Cell Content.

## Data Availability

No new data were generated or analyzed for this correspondence.

## References

[1] Nero C , Duranti S, Giacomini F, et al Integrating a comprehensive cancer genome profiling into clinical practice: a blueprint in an Italian Referral Center. J Pers Med. 2022;12:1746. 36294885 10.3390/jpm12101746 PMC9605534

[2] Mandelker D , Donoghue M, Talukdar S, et al Germline-focused analysis of tumour-only sequencing: Recommendations from the ESMO Precision Medicine Working Group. Ann Oncol. 2019;30:1221-1231. 31050713 10.1093/annonc/mdz136 PMC6683854

[3] Moore K , Colombo N, Scambia G, et al Maintenance olaparib in patients with newly diagnosed advanced ovarian cancer. N Engl J Med. 2018;379:2495-2505. 30345884 10.1056/NEJMoa1810858

[4] Daly MB , Pal T, Maxwell KN, et al NCCN guidelines^®^ insights: genetic/familial high-risk assessment: breast, ovarian, and pancreatic, version 2.2024. J Natl Compr Canc Netw. 2023;21:1000-1010. 37856201 10.6004/jnccn.2023.0051

[5] Tung N , Ricker C, Messersmith H, et al Selection of germline genetic testing panels in patients with cancer: ASCO guideline. J Clin Oncol. 2024;42:2599-2615. 38759122 10.1200/JCO.24.00662

[6] Klek S , Heald B, Milinovich A, et al Genetic counseling and germline testing in the era of tumor sequencing: a cohort study. JNCS JNCI Cancer Spectr. 2020;4:pkaa018. 10.1093/jncics/pkaa018 PMC7306190 32596633

[7] Mandelker D , Ceyhan-Birsoy O. Evolving significance of tumor-normal sequencing in cancer care. Trends Cancer. 2020;6:31-39. 31952779 10.1016/j.trecan.2019.11.006 PMC8923150

[8] Hayashi H , Kunimasa K, Tanishima S, et al Germline BRCA2 variant with low variant allele frequency detected in tumor-only comprehensive genomic profiling. Cancer Sci. 2024;115:682-686. 38086530 10.1111/cas.16043 PMC10859595

[9] Richards S , Aziz N, Bale S, et al Standards and guidelines for the interpretation of sequence variants: a joint consensus recommendation of the American College of Medical Genetics and Genomics and the Association for Molecular Pathology. Genet Med. 2015;17:405-424. 25741868 10.1038/gim.2015.30 PMC4544753

[10] Joynt ACM , Axford MM, Chad L, et al Understanding genetic variants of uncertain significance. Paediatr Child Health. 2021;27:10-11. 35273666 10.1093/pch/pxab070 PMC8900699

[11] He L , Zhang B, Yang J, et al A pitfall in targeted Sanger sequencing of BRCA splicing variants in at-risk individuals. Pathol Res Pract. 2021;222:153456. 33964677 10.1016/j.prp.2021.153456

